# Understanding Rehabilitative Education: Professionals’ Conceptions in Finnish Upper-Secondary Vocational Education

**DOI:** 10.5334/cie.435

**Published:** 2026-08-24

**Authors:** Anna-Kaisa Peruskivi, Anneli-Maarit Hermanoff, Tanja Äärelä

**Affiliations:** 1 University of Lapland, Finland

**Keywords:** Rehabilitative education, mental health, vocational education, upper secondary education

## Abstract

Mental health difficulties increasingly affect young people’s participation in education, creating new demands for educational support systems. In Finland, the concept of rehabilitative education has recently emerged in policy discussions as a potential response, particularly within upper-secondary vocational education, where support structures for students with mental health difficulties remain limited. This study adopted a qualitative phenomenographic approach to examine how professionals in upper-secondary vocational education conceptualise rehabilitative education. Based on eight focus group interviews with professionals (*N* = 41) working in vocational education and student welfare services, four qualitatively distinct but interconnected conceptions of rehabilitative education were identified: (a) supporting students’ individual resources, (b) pedagogical arrangements, (c) multiprofessional collaboration, and (d) structural solutions within the education system. Together, these conceptions form a multi-layered understanding that spans from individual support to broader institutional conditions. Interpreted through Amartya Sen’s capability approach and Nancy Fraser’s theory of social justice, the findings suggest that rehabilitative education can be understood as a way of expanding students’ opportunities to participate meaningfully in education. The study contributes to the emerging conceptualisation of rehabilitative education by clarifying its nature and its role in addressing mental health-related barriers to participation. Although situated in the Finnish context, the findings contribute to international discussions on how education systems can respond to students experiencing mental health difficulties.

## Introduction

Although precise prevalence data are lacking, it is estimated that approximately 20–25% of young people in Finland have a mental health disorder (Silén et al., 2025). Previous research shows that mental health difficulties impair learning (Gustafsson et al., 2010; Schulte-Körne, 2016), are associated with higher levels of absenteeism (Epstein et al., 2018; Finning, 2019), and increase the risk of dropping out of studies (Andersen et al., 2021; Askeland et al., 2022; Dupéré et al., 2018; Hjorth et al., 2016). From the perspective of education and social policy, this represents a significant challenge: Young people with mental health difficulties are more likely to become excluded from education and working life and consequently face a higher risk of broader social marginalisation (Ringbom et al., 2021).

The extension of compulsory education implemented in 2021 represented a major reform in Finnish upper-secondary education. Previously, young people were not legally required to continue their studies after completing basic education (comprising nine years of comprehensive schooling for children aged 7–16). With the Extended Compulsory Education Act (1214/2020), all young people are now obliged to continue their education until they complete a general or vocational upper-secondary qualification or reach the age of 18, whichever comes first. While this reform aimed to raise the overall level of education and strengthen equality, it increased pressure on student support systems, and not all young people have been able to find sufficient support to complete their upper-secondary studies (Jokio et al., 2024).

Integration of rehabilitative education into upper-secondary education has recently emerged in policy discussions in Finland as a potential solution to supporting students with mental health difficulties (Kuntaliitto, 2024). However, despite such increasing interest, rehabilitative education remains conceptually underdefined, making it difficult to develop coherent practices and structures to support students with mental health difficulties.

This study aimed to address this gap by examining how teaching, guidance, and student welfare professionals in upper-secondary vocational education conceptualise rehabilitative education.

In this study, rehabilitative education is understood provisionally as an approach that combines educational practices, psychosocial support, and multiprofessional collaboration to reduce mental health-related barriers to participation in education (e.g., Hoover & Bostic, 2021; Lövgren et al., 2020). As such, it is embedded in everyday educational settings and differs from clinical rehabilitation. Further, unlike special education, its primary emphasis is not on learning difficulties or formal pedagogical support but on enabling participation and continuity in education for students whose mental health difficulties hinder studying. We use the term *mental health difficulties* broadly to refer both to diagnosed mental health disorders and to other forms of psychological distress that impair students’ ability to study.

In the following, we first outline rehabilitative education in Finland and then present the theoretical framework and aim of the study. Next, we describe the phenomenographic methodology used and report the empirical findings. The article concludes with a discussion of the findings in relation to justice and realistic utopia, and their implications for practice and policy.

### Emergence of Rehabilitative Education in Finland

Rehabilitation has a long history in Finland, and its traditional forms are commonly divided into physical, psychological, social, and vocational rehabilitation. The field of rehabilitation also includes the notion of educational rehabilitation, which is closely linked to special education (Järvikoski, 2013; Tilus, 2018). In recent decades, approaches to rehabilitation have shifted from an emphasis on individual characteristics towards a more holistic understanding of functioning and the role of environmental factors, as reflected in frameworks such as the International Classification of Functioning, Disability and Health (ICF) (Finnish Institute for Health and Welfare, 2025; Järvikoski, 2013; Salminen & Häikiö, 2022). In the school context, this shift has expanded the focus from students’ individual abilities to the role of educational environments in supporting participation. For example, Puustjärvi and Luoma (2019) highlight how students’ capacity for school attendance is shaped not only by individual factors but also by the conditions provided by schools.

The term *rehabilitative education* is used in different ways in Finland. In basic education, it typically refers to hospital education, which ensures continuity of schooling during illness (Ruutu, 2019; Tilus, 2018). In addition, some municipalities have established rehabilitative small-group settings for students with mental health symptoms or socio-emotional support needs. However, these structures operate only at the level of basic education.

In upper-secondary education (which in Finland follows basic education and focuses on either academic or vocational qualifications for students aged 16–19), no equivalent rehabilitative structures currently exist within qualification-oriented education. Instead, the concept is primarily associated with intensive special support, particularly preparatory training for work and independent living (TELMA), which forms a part of young people’s broader rehabilitation (Finnish National Agency for Education, 2018). However, because TELMA is a non-qualification programme for students with disabilities or extensive support needs, it does not provide a rehabilitative framework for students pursuing formal vocational qualifications. This creates a significant gap in support for students with mental health difficulties, who are expected to complete qualification-oriented studies due to the extension of compulsory education.

### Theoretical Framework for Structuring Rehabilitative Education

In this study, rehabilitative education was examined specifically in the context of qualification-oriented vocational education, where the concept has not previously been clearly defined. Because the concept remains underdefined, theoretical perspectives are needed to clarify its meaning. We examined rehabilitative education from three complementary theoretical perspectives: Nancy Fraser’s theory of justice, the concept of realistic utopia, and Amartya Sen’s capability approach. These three theoretical perspectives each address different dimensions of equity in education. Together, they offer a lens through which rehabilitative education can be conceptualised.

#### Theory of Justice

Fraser’s (1998, 2008) theory of justice provides a structural starting point for assessing how the education system can support or limit participation of young people with mental health difficulties. Justice in education is based on the redistribution of resources, the recognition of different needs, and the opportunity for participation (representation) (Fraser, 1998, 2008; Keddie, 2012; Mills et al., 2015). In the context of education, emphasis is also placed on affective justice – care, emotional support, and belonging – as well as contributive justice, which refers to the opportunity to participate in meaningful activities (Lynch, 2012; Mills et al., 2016; Sayer, 2009, 2011).

#### Realistic Utopia

The concept of realistic utopia refers to a social ideal that is both desirable and, in principle, attainable. It is therefore not a utopian dream, but a social model grounded in actual institutional possibilities (Rawls, 1999). Fraser (2008) developed the concept of realistic utopia as a critical theoretical tool that helps to identify existing injustices as well as the institutional possibilities for overcoming them. In this study, the idea of a realistic utopia was used to support reflection on what kinds of pedagogical and structural changes could make the education of young people with mental health difficulties more equitable.

#### Capability Approach

Sen’s (1993, 2009) capability approach complements Fraser’s structural level and brings the individual’s perspective to the analysis. It reminds us that the significance of education is not limited to formal access to studies but is linked to the actual opportunities young people have to build a meaningful future based on their own starting points. The approach has also been applied in the contexts of education and special needs support (Terzi, 2005, 2008), which makes it a particularly relevant framework for examining the support of young people with mental health difficulties.

Together, these three perspectives connect individual and structural levels of analysis and provide a framework for interpreting rehabilitative education. While our phenomenographic analysis focused on describing qualitatively different ways of understanding rehabilitative education, these theoretical perspectives were used in the interpretative phase to examine the broader educational and societal implications of the identified conceptions.

### The Present Study

The study adopted a qualitative phenomenographic approach, which is particularly suited to identifying qualitatively different ways of understanding a phenomenon and examining the relationships between these conceptions at a collective level (Ashworth & Lucas, 1998; Marton, 1981). Accordingly, we sought to identify and describe the variation in professionals’ conceptions of rehabilitative education. The resulting descriptive categories form the basis for a theoretically informed interpretation drawing on perspectives of educational justice and the capability approach. The study addressed the following research question.

How do professionals in Finnish upper-secondary vocational education conceptualise rehabilitative education as a form of support for students with mental health difficulties?

## Method

### Participants and Setting

The data were produced through focus group interviews. Participants were recruited through 22 vocational institutions (i.e., upper-secondary institutions providing qualification-oriented vocational education), eight wellbeing services counties (regional authorities responsible for organising public health and social services), and three vocational special education institutions that had granted research permission.^1^ Participation was based on voluntary registration following an open invitation. All participants provided informed consent electronically prior to the interviews.

The resulting dataset was diverse, including professionals from different types of institutions and regions. In total, 41 professionals participated in eight separate focus group interviews, each consisting of four to six participants. The participants represented various professional roles within education, guidance counselling, and student welfare services, as summarised in Table 1.

**Table 1:** Participants’ Professional Backgrounds.


PROFESSIONAL GROUP	PROFESSIONAL ROLES	*N*

Teaching Staff	Vocational teachers, special education teachers, vocational special education teachers, preparatory education (TUVA) teachers, teachers of common units	25

Guidance & Welfare Staff	Study counsellors, school social workers, school psychologists, student welfare coordinators	9

Instructional Support Staff	Vocational assistants, workshop assistants	4

Administration & Management	Education managers, directors of development, workshop coaching managers	3


Focus group interviews were used to elicit both individual perspectives and interactive discussion (Williams & Katz, 2001). The interviews were conducted in August–September 2025 via Microsoft Teams. Each interview lasted 75 minutes. All sessions were audio-recorded using an external recording device and subsequently transcribed verbatim. A semi-structured interview guide was used consistently across all focus group interviews, directing the discussion towards the perceived need for rehabilitative education, its practical implementation, and the opportunities and challenges related to its development.

### Data Analysis

The analysis followed established principles of phenomenography (see Ashworth & Lucas, 1998; Marton, 1981; Åkerlind, 2005, 2025). Conducted primarily by the first author, the analysis proceeded in several iterative stages. First, the transcripts were read multiple times to achieve familiarity with the data. Second, meaningful expressions, sentences, and units of thought were identified. These were then compared and grouped based on similarities and differences. Third, descriptive categories were constructed to represent the qualitatively different ways in which rehabilitative education was understood (Åkerlind, 2025).

The development of categories was iterative and involved continuous comparison between data extracts and emerging categories. The preliminary categories were discussed and refined in collaboration with the research group, as part of ensuring the credibility and trustworthiness of the phenomenographic analysis. The structural relationships between categories were also examined to capture the overall variation in conceptions. Analytical saturation was considered reached when no new qualitatively different conceptions emerged in the final focus group interviews. Finally, the descriptive categories were presented in a condensed and structured form. The categories do not describe individual participants’ fixed opinions. Instead, they represent qualitatively different ways in which rehabilitative education was conceptualised across the data.

## Results

Based on the results of the focus group interviews, rehabilitative education is described through four descriptive categories that represent different ways of understanding its nature and its role in vocational education: supporting students’ individual resources, pedagogical arrangements, multiprofessional collaboration, and structural solutions.

### Individual Level – Rehabilitative Education as Supporting Students’ Individual Resources

At the individual level, rehabilitative education was seen as centred on responding to students’ individual support needs. Participants emphasised the importance of recognising and working with the young person’s available resources, which often means that goals need to be very small. Participation was viewed as more important than academic progress; for example, simply arriving at school or attending a lesson was interpreted as a sign of recovery.

The emphasis was on managing daily life and supporting endurance, and temporarily stepping back from qualification-oriented expectations was considered justified. This emphasis on lowering expectations is reflected in the following quotation: “There wouldn’t need to be any goal other than coming to school and doing something. And then, when the student has more strength, they could return to more goal-oriented work.” (Group 5, vocational teacher)

In planning support, participants also emphasised recognising the young person’s symptoms and their underlying causes. Participants described mental health difficulties such as depression, fear of social situations, and generalised anxiety, which were reflected in everyday life as absenteeism, reduced functioning, and difficulties participating in group situations. Absences, in turn, slow academic progress, weaken peer relationships, and make it more difficult to provide support. Nevertheless, every young person’s right to study was understood as a foundation for developing rehabilitative education. The key starting point is recognising the core of the issue, as one of the interviewees explained: “It’s about investing in finding the core of the issue. Individually, for each student. Which string, or which combination of strings, you need to pull to help them move forward.” (Group 6, school social worker)

The target group for rehabilitative education was seen to include, for example, young people who have not received group-based instruction in basic education. For them, the transition to vocational education is a major step, and they need a small and safe group in which to practice interaction and study skills. The same applies to students transitioning from hospital education, returning from medical leave, or those for whom a placement in a vocational special education institution would be necessary but unavailable. Participants emphasised that the young person’s difficulties should be recognised as requiring targeted support rather than being viewed as permanent barriers.

The participants described the core task of rehabilitative education as strengthening the young person’s personal resources. This involved highly individual pathways and progressing in small steps. Experiences of success were seen as essential factors that enhance a student’s sense of capability. Rehabilitative education was understood as something that must be built upon the student’s own needs rather than implemented through predetermined models.

Establishing a trusting relationship was seen as central to providing support, and this was portrayed as a long-term process. The importance of sufficient continuity was emphasised, as recovery takes time. Both the young person and their guardians were viewed as experts in the student’s support needs and recovery, and their active involvement in planning was regarded as crucial. This individualised orientation was further emphasised by one participant: “There isn’t just one way. Every individual, every student has their own challenges and their own special support needs. You can’t place anyone in a fixed mould.” (Group 2, vocational special education teacher)

In sum, in this descriptive category, rehabilitative education was seen primarily as support for the student’s everyday life and wellbeing. As such, participants noted that pedagogical solutions, multiprofessional collaboration, and structural changes only gain meaning in relation to the young person’s personal situation and daily life.

### Pedagogical Level – Rehabilitative Education in Terms of Pedagogical Arrangements

At the pedagogical level, rehabilitative education was understood as maintaining a balance between supporting the process of rehabilitation and the structural features of formal schooling, as described in this quotation: “When we talk about rehabilitative education, we also mean keeping some degree of ‘school-likeness.’ So that the step from that environment back into a school environment isn’t too big.” (Group 6, school psychologist)

Rehabilitative education was also understood as the organisation of teaching and the learning environment. That is, its goal is to provide pedagogical solutions that enable participation for young people experiencing mental health difficulties and to reduce the overall strain of schoolwork. Small groups were highlighted as a key practice: They allow for more individual attention and reduce social pressure. Participants also underscored the importance of avoiding excessive heterogeneity when forming such groups, so that students can benefit from peer support.

Calm and clearly structured spaces were seen as essential, as they help reduce sensory overload and promote psychological safety. Participants expressed a wish for spaces that feel more homelike and differ from traditional classrooms, offering opportunities for both hands-on, activity-based learning and access to private, quieter areas when needed.

Although small groups were widely supported, their potential isolating effect was also recognised. In addition, small groups were understood as insufficient for meeting the needs of all students who require support. For this reason, there was an expressed need to integrate support measures into the everyday activities of mainstream groups as well, as illustrated in the following excerpt: “Perhaps in an ideal situation, we would have enough support within the mainstream programmes and for the teachers there, so that most students could be supported in that environment. And then, for those who still cannot manage there, rehabilitative education would be available.” (Group 6, study counsellor)

This way of understanding rehabilitative education also includes flexible schedules and the possibility of reducing the study load so that students can progress at their own pace without excessive pressure. This is reflected, for example, in the idea of having an “empowerment year” before TUVA studies (TUVA is a preparatory programme designed to strengthen students’ basic study skills before entering general or vocational upper secondary education). During this empowerment year, the student would be given time to build personal resources before moving on, as one participant described: “A TUVA year is essential, but many 15-year-olds would actually need two years. First, a rehabilitative year, and then the TUVA year, where they practise work life skills, study skills, complete common studies, visit different vocational programmes, and so on. But before that, the empowerment year.” (Group 7, preparatory education teacher)

Pedagogical arrangements were understood as means of providing small experiences of success through which students gradually strengthen their belief in their ability to learn. At the same time, participants described teaching as needing to remain purposeful and to support students’ progress, even if this requires stepping slightly outside one’s own comfort zone. The young person should actively participate in setting their own goals. Progress can be supported by positive feedback, which should be given even for the smallest steps. As one participant noted, studying itself can be rehabilitative: “At its best, studying itself is rehabilitative. When a young person starts to get a grip on their life and a positive cycle begins.” (Group 5, vocational assistant)

The content of teaching was described as emphasising emotional and interaction skills, the pedagogical integration of everyday life activities, and activity-based learning. During the rehabilitative period, students may also complete portions of their qualification, enabling their study path to progress. Rehabilitative education was thus seen as part of the student’s studies but implemented in an environment that is easier to cope with. At the centre of pedagogical support are trusting teacher-student relationships, which require time and genuine encounters to develop. In-person teaching was considered important, as it enables a sense of community, the development of peer relationships, and the building of support structures. Participants described the aim as supporting the student in coming to school and in forming a connection with the institution. As one participant expressed: “If the student would even come to school, there is already so much one can start doing at that point.” (Group 5, vocational special education teacher)

In sum, in this descriptive category, rehabilitative education was understood primarily as pedagogical arrangements that create a flexible and safe learning environment. The focus was not on the young person’s symptoms, the coordination of services, or systemic conditions, but on adapting teaching so that a student experiencing mental health difficulties can participate and experience success.

### Multiprofessional Level – Rehabilitative Education as Multiprofessional Collaboration

At the multiprofessional level, rehabilitative education was understood primarily as network-based collaboration in which no single professional is sufficient. It was emphasised that teachers cannot bear responsibility for supporting a young person experiencing mental health difficulties on their own; as one participant noted: “It cannot function solely through the work of teaching staff.” (Group 8, preparatory education teacher)

Instead, participants described a broad and closely connected multiprofessional network as necessary and emphasised that rehabilitative education requires a seamless connection between teaching and other services. The necessity of strong collaboration with healthcare was expressed as follows: “If rehabilitative education were to be organised, there would need to be very strong collaboration with the healthcare providers. When we talk about rehabilitation, it cannot be rehabilitation if it is carried out solely among the actors of the education sector. The link to healthcare must be genuinely seamless.” (Group 7, school social worker)

Key actors identified included student welfare professionals as well as the young person’s wider network, such as adolescent psychiatric services and social work. Multiprofessional collaboration also referred to cooperation with workshop activities, coaches, and other parallel services that work alongside the education sector. The involvement of families was likewise considered important. Participants described networks as essential for supporting the student holistically, both in their studies and in everyday life.

Integrating services into the student’s educational pathway was seen as crucial, as one participant noted: “It could really be in the student’s best interest if the services were located here at the school, or at least strongly integrated into the school day.” (Group 5, preparatory education teacher)

Also, a trusted adult who accompanies the young person when navigating different services and ensures continuity of support was considered important. Further, the value of co-working practices was highlighted. These bring multiprofessional collaboration into the everyday life of the school. Suitable co-working partners for teachers were envisioned to include psychiatric nurses or social counsellors. The need for multiprofessional collaboration was further described in the following excerpt: “There are the workshops, but they are a separate environment. Then there are the educational institutions. And then there is healthcare. But we need something that connects these, because the phenomenon is so broad and the need is so clear.” (Group 8, preparatory education teacher)

Across the discussions, a recurring concern related to the distribution of responsibility. Educational institutions were perceived as carrying an excessive share of responsibility for the young person’s rehabilitation. This situation was experienced as burdensome, highlighting the need for clearer divisions of responsibility and more permanent structures for collaboration. Participants linked successful rehabilitative education to continuous information flow and the formation of a shared understanding of the student’s situation. At the centre of multiprofessional work was the young person and their needs, as stated in this quote: “How do we do what is best for this young person, each of us from our own perspective?” (Group 1, special education teacher)

In sum, in this descriptive category, rehabilitative education was understood primarily as multiprofessional collaboration in which the expertise of different professionals is brought together to support the student. The emphasis was on coordinating networks and sharing responsibility so that a coherent and continuous system of support can be built around the young person.

### Structural Level – Structural Solutions in Rehabilitative Education

At the structural level, participants described rehabilitative education as a response to systemic gaps within the education system. As one participant observed: “The same kinds of concepts are being invented in different parts of Finland simply because people have noticed that there is a missing piece in the system.” (Group 6, student welfare coordinator)

Rehabilitative education was viewed as part of the development of the education system and its structures. In this view of rehabilitative education, individual pedagogical solutions or multiprofessional collaboration are not sufficient if system-level practices and constraints prevent the provision of support for young people experiencing mental health difficulties. Participants raised concerns related to fairness and equity within the education system and described current structures as leaving some young people without adequate support.

The main challenges identified included the current constraints of the Extended Compulsory Education Act and funding models, the demanding everyday life of large campus settings, and the limited availability of suitable learning environments. The Extended Compulsory Education Act was seen as requiring young people to study even when they may not have the functional capacity to do so. The existing funding model was considered inflexible because it incentivises the rapid completion of qualifications and does not take into account the slow and uneven processes of rehabilitation. Young people with mental health difficulties were not seen to have a clearly defined place within the education system, as one interviewee commented: “Somehow our society has created a situation where young people who need one or two years to recover simply don’t have a place.” (Group 7, vocational special education teacher)

The existing structure of the TUVA was seen as a potentially suitable option for students experiencing mental health difficulties. From the perspective of pedagogical support, the TUVA curriculum was regarded as broad and flexible, allowing for different emphases. However, its challenges include group sizes that were considered too large, limited opportunities for individualised support, and insufficient staffing resources. As a solution, developing small-group forms of TUVA in which the group would be guided by a teacher–nurse pair was suggested.

All institutions represented in the focus groups had developed some form of local solution to support students with mental health difficulties. However, the need for national guidelines and long-term resources was emphasised, so that effective local practices do not remain short-term project-based initiatives or vary significantly between regions. One of the participants noted: “We need to plan with a long-term perspective. Right now, everything is very project-based. You develop a model for two years, the project ends, and it disappears. Then a new one is invented. We need continuity – development that really leads to making the work function well. And practices shouldn’t differ so much from one place to another.” (Group 6, school psychologist)

Participants also highlighted the need to safeguard staff wellbeing and endurance. Rehabilitative education was seen as requiring specialised expertise and long-term commitment, which cannot be achieved without sufficient resources. For this reason, a national mandate and clear policy guidelines were considered essential. With regard to resources, it was also noted that, in the long term, rehabilitative education may reduce societal costs by preventing young people from becoming marginalised. These concerns were articulated clearly by one participant: “I think rehabilitative education is important right now so that we can achieve the goals of extending compulsory education: that young people don’t become excluded and stay at home. With the means we currently have, we will never reach that goal. Rehabilitative education could be one solution.” (Group 7, preparatory education teacher).

In sum, in this category, participants described rehabilitative education as having potential long-term societal benefits, including prevention of marginalisation. Participants emphasised the need for creation of permanent structures that guarantee access to individualised support, pedagogical solutions, and multiprofessional collaboration equally across the country.

## Discussion

This section examines the findings of the study by first summarising the phenomenographic results. The discussion develops this understanding further by conceptualising rehabilitative education as a realistic utopia, drawing on theories of social justice. Finally, implications for practice and policy are presented.

### Summary of the Findings

**Figure 1:**
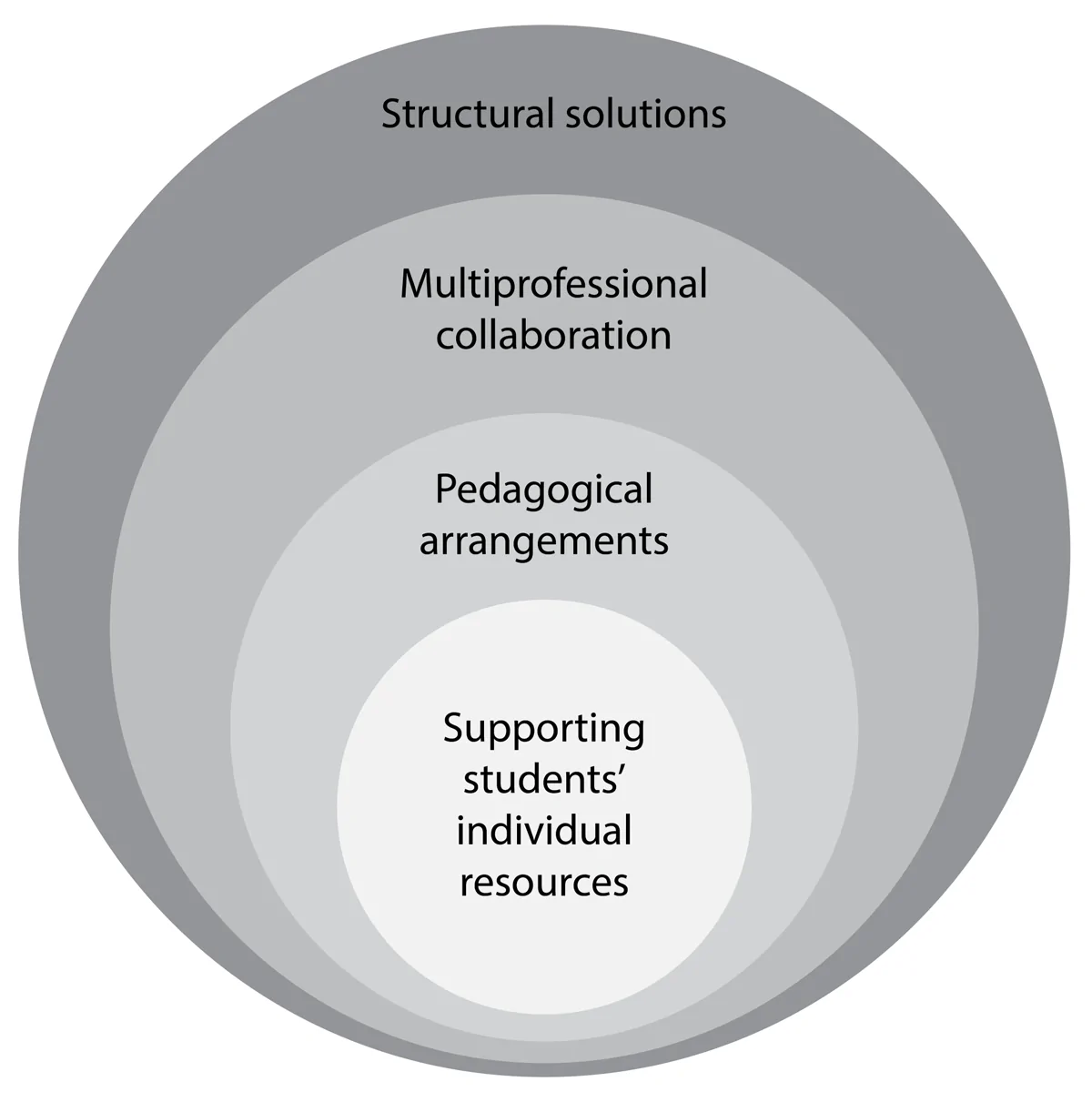
Conceptions of Rehabilitative Education Identified Through Phenomenographic Analysis.

The descriptive categories showed that the professionals in our focus groups viewed rehabilitative education as a multi-layered phenomenon structured across four complementary levels. These levels are not mutually exclusive; rather, they are interconnected. Support for the student’s personal resources is enacted through pedagogical arrangements, while multiprofessional collaboration provides continuity around these practices, and structural solutions create the conditions under which they can be realised. Together, these levels form a comprehensive understanding of rehabilitative education both as a set of practical activities and as an educational policy investment. This conceptualisation is illustrated in Figure 1.

While Figure 1 illustrates the overall structure of rehabilitative education as a multi-layered phenomenon, Table 2 summarises the core elements that characterise each level and highlights their analytical distinctions. The levels and elements of rehabilitative education identified in this study can also be recognised in international models and school-based interventions. For example, research has shown that strengthening individual resources, adapting pedagogical practices, and promoting multiprofessional collaboration can support the educational trajectories of young people experiencing mental health difficulties (e.g., Cooper & Whitebread, 2007; Lövgren et al., 2020; Richter et al., 2022). Similarly, previous research emphasises the importance of delivering support services within young people’s everyday environments, such as schools (Heinonen, 2024; Hillborg et al., 2021; Hoover & Bostic, 2021). This study extends these perspectives by demonstrating that questions of justice are also embedded in the structural conditions of education systems.

**Table 2:** Core Elements of Rehabilitative Education.


LEVEL	CORE ELEMENTS

Individual level	Responding to individual needs Strengthening personal resources Ensuring continuity of support

Pedagogical level	Safe and structured learning environment Small-group instruction Realistic and attainable study goals

Multiprofessional level	Clear division of responsibilities and coordination The student at the centre of multiprofessional support

Structural level	Adequate resources Equal access to support across regions


### Findings Seen Through the Lens of Justice

From the perspective of Amartya Sen’s (1993, 2009) capability approach, the value of education cannot be assessed solely through formal participation, but through the actual opportunities and freedoms it provides for young people. In this study, justice was understood as the extent to which education systems enable young people’s real opportunities to participate in education and to progress in ways that are meaningful for them. The findings illustrate that injustice may arise from a lack of individual support. In addition, it may arise from structural conditions such as inflexible funding models, large institutional settings, and limited availability of suitable learning environments. From this perspective, rehabilitative education makes visible how questions of justice are interconnected in everyday educational practices.

While the findings emphasize structural challenges related to legislation, resources, and service coordination, they also point towards a more fundamental dimension of rehabilitative education. That is, beyond policy and system-level solutions, participants’ descriptions highlight the importance of recognising young people as individuals with unique needs, vulnerabilities, and capacities. In this sense, rehabilitative education can also be understood as an ethical and relational practice.

The findings of this study suggest that *rehabilitative education* is not merely another term for special or inclusive education. Rather, it reflects a distinct support orientation that emerges at the intersection of pedagogy, psychosocial support, multiprofessional collaboration, and structural conditions. Its added conceptual value lies in making visible how mental health-related barriers to participation are addressed not only through teaching arrangements, but also through relational, institutional, and systemic forms of support. From this perspective, context can be understood as the space in which barriers to and facilitators of participation are actively produced and negotiated. In this sense, rehabilitative education does not only respond to individual needs. It also involves transforming the conditions within which participation becomes possible.

### Rehabilitative Education as a Realistic Utopia

Our findings can also be understood through the concept of a realistic utopia. Rehabilitative education is still an emerging and partly contested idea, yet the professionals in this study view it as a necessary response to current challenges. In this context, a realistic utopia refers to envisioning solutions that are grounded in present realities but also exceed existing structures and open possibilities for new ways of organising educational support (Fraser, 2008; Rawls, 1999).

The dimensions of justice provide a conceptual tool for outlining such a realistic utopia. Table 3 summarises how the dimensions of justice are reflected in the data when viewed through this lens. The examples identified in the analysis span different levels of the education system: Some relate to structures and the distribution of resources, while others concern everyday practices and students’ lived experiences. By framing rehabilitative education as a realistic utopia, the analysis highlights its potential not only to address immediate gaps in support but also to reimagine how educational systems may respond to the needs of students experiencing mental health difficulties in more just and enabling ways.

**Table 3:** Dimensions of Justice in Rehabilitative Education as a Realistic Utopia.


DIMENSION	HOW REHABILITATIVE EDUCATION IS CONSTRUCTED	EXAMPLE CITATION

Redistribution	Small groups, flexible study pathways, and sufficient support services enable individualised support.	“That we would have time to build those individual solutions — and the resources to actually implement them in practice.” (Group 3, special education teacher)

Recognition	Mental health difficulties are recognised as part of human diversity. Each student’s individual needs are acknowledged and responded to.	“An attitude where we see everyone in our society as capable and valuable — even if at this moment someone is struggling.” (Group 7, vocational special education teacher)

Representation	The student and their network participate in decision-making. The student is at the centre of multiprofessional collaboration.	“That the young person can help decide their goals and what they start working towards.” (Group 3, vocational assistant)

Affective justice	The learning environment is psychologically safe. Emotional support is sufficient.	“There must be enough adults who have the time to meet the student and be present.” (Group 6, workshop coaching manager)

Contributive justice	Students have opportunities to participate in the community. Study goals generate experiences of success.	“That they could do something genuinely meaningful to them — and then feel those moments of success.” (Group 1, special education teacher)


From the perspective of Fraser’s (1998, 2008) theory of justice, rehabilitative education involves redistribution of resources, recognition of students’ needs, and their opportunities for participating in decisions concerning their educational pathways. The data also repeatedly highlight the importance of emotional support and a sense of belonging, which relates to the concept of affective justice (Lynch, 2012; Mills et al., 2016). In addition, the professionals emphasised the importance of students feeling valued and able to contribute meaningfully, which can be understood through the lens of contributive justice (Mills et al., 2016; Sayer, 2009, 2011).

Viewed as a realistic utopia, rehabilitative education emerges as a vision of an educational system in which every young person has genuine capabilities for action. Positioned in this way, the concept provides a constructive framework to consider how education systems might evolve to better support students whose needs challenge conventional structures. This study contributes to existing research by offering a multilevel conceptualisation of rehabilitative education that explicitly links individual, pedagogical, multiprofessional, and structural dimensions of support.

### Implications for Practice

The findings of this study suggest that rehabilitative education can be understood not only as a set of support measures but also as an ethical and relational practice. For educators and student welfare professionals, this view implies a shift in focus from academic performance alone towards recognising students’ current functional capacity as a valid starting point for learning.

In practice, this may involve prioritising participation and presence over immediate academic outcomes, particularly in situations where students are experiencing significant mental health difficulties. Creating pedagogical arrangements that reduce pressure and enable participation is, therefore, essential. This includes, for example, the use of small-group settings, calm and clearly structured environments, and spaces that differ from traditional classrooms in ways that promote psychological safety.

The findings also highlight the importance of integrating support into everyday school practices. Rather than relying solely on referrals to external services, practitioners may benefit from developing co-working models in which educational and mental health professionals work alongside each other within the school environment. Such approaches can strengthen continuity of support and reduce fragmentation in service provision.

Our findings also have implications for the competencies required of professionals working within rehabilitative education. A more detailed description of the competencies, training, and areas of expertise associated with this role is provided in the Supplementary File 1, where the profile of a rehabilitative educator is outlined.

### Implications for Policy

The findings of this study indicate that the development of rehabilitative education requires coordinated policy responses at multiple levels of the education system. At the national level, the results highlight the need for clearer policy frameworks and long-term structural solutions. This includes the development of national guidelines and a potential legal mandate for rehabilitative education to reduce regional variation. In addition, current funding models may need to be reconsidered, as they tend to prioritise rapid completion of qualifications and may not adequately take into account the slower and non-linear processes of recovery described in the data.

At the institutional level, the findings suggest a shift from short-term, project-based initiatives towards more permanent structures within educational organisations. This may involve allocating dedicated staff, developing specialised pedagogical environments, and embedding rehabilitative practices within mainstream vocational education settings.

The results also underline the importance of strengthening coordination between education and healthcare systems. Policies that support integrated service provision and clarify the distribution of responsibility between sectors may help to reduce fragmentation and ensure continuity of support for young people with mental health difficulties.

## Conclusion

This study explored how professionals conceptualise rehabilitative education in Finnish upper-secondary vocational education. Through focus group interviews, four qualitatively different ways of understanding this approach were identified. The findings show that rehabilitative education constitutes a multilayered whole that brings together pedagogical arrangements, multiprofessional collaboration, and structural conditions that respond to the student’s individual needs. The professionals’ accounts also highlighted key structural challenges, such as the qualification-oriented funding model and the lack of suitable learning environments, that limit young people’s capabilities. Taken together, these insights demonstrate that rehabilitative education is best understood as a structural and relational approach rather than an individual-level intervention.

From a societal perspective, the results of the study underscore the reality that supporting young people with mental health difficulties cannot be the responsibility of individual schools or professionals alone. A structural solution is needed. Rehabilitative education can be understood as a realistic utopia: an ambitious yet feasible investment in social justice, preventing marginalisation and reinforcing the sustainability of the welfare state. Positioned in this way, the concept offers a framework that may also inspire reflection in other education systems seeking more just and enabling responses to the needs of students whose mental health challenges place them at the margins of conventional schooling.

### Limitations of the Study

This study has certain limitations. Specifically, it was based on professionals’ conceptions rather than observed practices. Participation was voluntary, which may have influenced the perspectives represented. In addition, the study focused on the Finnish education system, which may limit the transferability of the findings to other contexts.

### Implications for Further Research

If rehabilitative education is developed as a part of the vocational education system, future research should examine how it is experienced by young people themselves through qualitative studies. Longitudinal and practice-based research is also needed to investigate how rehabilitative education is implemented in practice and how it influences educational pathways and wellbeing over time. Comparative international studies could further deepen understanding of how similar challenges are addressed in different educational systems.

## Use of Artificial Intelligence

An AI-based writing assistant (Grammarly) was used to identify grammatical issues and improve sentence clarity. All suggested revisions were carefully reviewed by the authors, who ensured the accuracy and originality of the manuscript. No research content, data, analyses, or references were generated using AI.

## Additional File

The additional file for this article can be found as follows:

10.5334/cie.435.s1 Supplementary File 1. Professional Profile of a Rehabilitative Educator.

## Footnotes

In this context, *research permission* refers to institutional approval to access participants as well as compliance with relevant ethical and data protection requirements. The research permissions covered a wide range of educational environments across Finland, with the exception of South Karelia and the Åland Islands.
