# Supplementary material for: Understanding Rehabilitative Education: Professionals’ Conceptions in Finnish Upper-Secondary Vocational Education
Source: Contin Educ. 2026 Aug 24;7(1):67–81. doi: 10.5334/cie.435 (PMC13523808; doi:10.5334/cie.435)
Supplement: Supplementary file 1 [file cie-7-1-435-s1.pdf]

Understanding Rehabilitative Education:
Professionals' Conceptions in Finnish Upper-Secondary Vocational Education

Supplementary Material 1

Professional Profile of a Rehabilitative Educator

Anna-Kaisa Peruskivi, Anneli-Maarit Hermanoff, Tanja Äärelä

Introduction

Schools are increasingly recognised as key contexts for mental health promotion, identification, and intervention, forming a central component of the broader child and adolescent mental health system (Hoover & Bostic, 2021). At the same time, implementing school-based mental health support is complex and requires coordination across educational, health, and social sectors (Richter et al., 2022). In Finland, rehabilitative education has recently emerged in policy discussions as a potential approach to supporting students with mental health difficulties in upper-secondary education. This discussion raises the question of who can be considered a rehabilitative educator and what kinds of competencies such a role requires.

This Supplementary File presents a proposed professional profile of a rehabilitative educator, based primarily on the findings of the present study and supported by previous research. It is intended to support the practical interpretation of the findings reported in the main article. While the profile is informed by broader theoretical frameworks, the competencies outlined here are derived from the empirical findings of this study and further contextualised through practice-oriented research on teacher competence and inclusive education. As rehabilitative education is an emerging concept, the profile is not presented as a fixed or prescriptive model but as an analytically grounded framework that makes visible the types of competencies required to support students with mental health difficulties.

Previous research has conceptualised teacher competence as a combination of knowledge, skills, and attitudes (see, e.g., Abdullah & Rashid, 2024; Kazanowski et al., 2025; Zakaria et al., 2024), aligning with broader models of professional competence (Metsäpelto et al., 2022). In the current profile, competence is understood as the capacity to act effectively in complex and context-sensitive educational situations. The concept of mental health literacy (MHL) has similarly been used to describe the competencies needed to recognise mental health difficulties and support help-seeking (Prabhu et al., 2025; Suwanwong et al., 2024). From the perspective of the findings of this study, rehabilitative education extends beyond MHL towards a broader, pedagogically grounded orientation that emphasises participation, continuity, and the reduction of mental health-related barriers to learning (e.g., Hillborg et al., 2021).

The competencies outlined in this profile are consistent with previous research on effective teaching across educational contexts (see, e.g., Iahn-Aun et al., 2025; Paakkari et al., 2024; Woolf, 2018), while also reflecting the specific demands of supporting students whose educational participation is shaped by psychological distress and fluctuating functional capacity. In addition to identifying core competencies, the profile aims to support conceptual clarity and responsible knowledge translation by making explicit the expectations related to professional preparation, collaboration, and ongoing development in this emerging field of practice.

The original article can be downloaded from https://doi.org/10.5334/cie.435

Core Competencies of a Rehabilitative Educator

Competence Area	Core Competencies and Practical Implications	Supporting References
**Pedagogical flexibility**	Ability to design flexible and adaptive learning pathways that account for students' fluctuating functional capacity and mental health-related barriers to participation. This includes breaking study goals into manageable steps, adjusting pacing, and supporting gradual engagement through attainable learning tasks. It also involves creating psychologically safe learning environments characterised by predictability, clarity, and reduced cognitive and emotional load (e.g., small-group settings). In addition, flexible assessment practices and alternative ways of demonstrating competence enable students to experience success and build confidence in learning.	Metsäpelto et al. (2022)<br>Paakkari et al. (2024)<br>Zakaria et al. (2024)
**Mental health literacy**	Knowledge and skills to recognise psychological distress and understand its impact on learning, participation, and everyday functioning. This includes the ability to identify the core of the issue and to interpret behaviours (e.g., absenteeism) as expressions of underlying needs rather than isolated problems, and to use this understanding as a basis for planning appropriate support. It also involves reducing stigma and supporting appropriate help-seeking without assuming a clinical role.	Prabhu et al. (2025)<br>Suwanwong et al. (2024)
**Relational and affective competence**	Ability to build and sustain trusting relationships and create a sense of psychological safety, belonging, and emotional support. This involves sensitivity to students' lived experiences and the ability to respond in ways that strengthen trust, recognition, and connection. It includes providing constructive, nonstigmatising feedback that supports students' agency and participation in situations of vulnerability, as well as fostering hope and future-oriented thinking. Appropriate professional boundaries and nonreactive responses support the development of a stable and safe relational context.	Calandri et al. (2025)<br>Fernandes et al. (2021)<br>Äärelä et al. (2016)
**Multiprofessional collaboration**	Ability to work across professional boundaries and integrate pedagogical, social, and healthcare perspectives. Multiprofessional collaboration is a necessary condition for supporting students with mental health difficulties and requires the ability to act as a bridge between education and support services. In practice, this involves coordinating support processes, communicating with guardians, and supporting students in accessing appropriate services, while maintaining a shared direction across different actors. It also includes recognising the limits of one's own professional role and working within appropriate boundaries in collaboration with other professionals.	Anvik & Waldahl (2018)<br>Fernandes et al. (2021)<br>Lövgren et al. (2020)
**Professional orientation**	Willingness to work in conditions of uncertainty, slow progress, and non-linear development, combined with a commitment to long-term support processes. This involves recognising that progress may be partial, uneven, and difficult to measure, and understanding the educator's role in supporting students' participation and the development of their capabilities. It reflects a shift from deficit-based interpretations towards a more contextual and relational understanding of students' situations, as well as a solution-focused approach that emphasises strengths, possibilities, and emerging capabilities.	Calandri et al. (2025)<br>Fernandes et al. (2021)<br>Hillborg et al. (2021)<br>Metsäpelto et al. (2022)

**Continuous professional development**	Ability to reflect on one's own pedagogical and relational practice, combined with a commitment to continuous learning and professional growth. This includes critical thinking, problem-solving, and metacognitive awareness, enabling educators to adapt their practice to complex and evolving situations. It also involves recognising the emotional demands of the work, maintaining professional wellbeing and resilience, and engaging in structured forms of professional development such as training, coaching, and collaborative learning processes.	Dinnen et al. (2024) Iahn-Aun et al. (2025) Paakkari et al. (2024)
**Structural advocacy**	Awareness of how institutional practices, policies, and resource structures shape students' opportunities to participate meaningfully. The ability to identify and challenge systemic barriers, while recognising the structural and professional conditions that define the scope of educators' roles. In practice, this may involve advocating for flexible study pathways, facilitating access to support services, and contributing to the development of more inclusive institutional practices.	Lawson et al. (2025) Lövgren et al. (2020) Manjari & Srivastava (2020)

Concluding Remarks

Proposed competencies do not operate independently but form an interrelated framework. The proposed profile highlights that supporting students with mental health difficulties is a holistic task that extends beyond traditional teaching roles (e.g., Lövgren et al., 2020). It requires not only specific competencies but also a rehabilitative orientation as outlined in this profile. Teaching is an inherently complex and demanding profession requiring expertise that is continuously developed, refined, and renewed (Metsäpelto et al., 2022). From the perspective of rehabilitative education, this highlights the importance of ongoing professional development in contexts characterised by uncertainty, slow progress, and nonlinear learning trajectories.

While the proposed profile does not define a new professional title, it raises important questions regarding training, qualification, and professional standards. The findings of this study suggest that rehabilitative education requires a combination of formal teacher education and additional competencies related to mental health, psychosocial support, and multiprofessional collaboration. In practice, this may involve initial teacher education (e.g., special education teacher qualification), complemented by training in mental health literacy, as well as structured opportunities for interdisciplinary learning and collaboration.

At present, no formal accreditation system exists for rehabilitative educators. However, articulating competencies may support the development of shared standards and guidelines, helping ensure that the concept is not applied in practice without adequate preparation. Rather than defining a new profession, this profile conceptualises rehabilitative education as a field of practice requiring hybrid expertise. That is, supporting students with mental health difficulties often requires multiprofessional collaboration, as a single professional cannot address these complex needs alone. Further research is needed to examine how proposed competencies can be developed, supported, and embedded within existing educational systems.

References

Abdullah, N., & Rashid, S. (2024). Vocational special education teachers' competencies: A literature review. *International Journal of Academic Research in Progressive Education and Development, 13*(3), 1876–1885. http://dx.doi.org/10.6007/IJARPED/v13-i3/22174

Anvik, C., & Waldahl, R. H. (2018). Sustainable collaboration to support vulnerable youth: Mental health support teams in upper secondary school. *Social Inclusion, 6*(3), 282–288. https://doi.org/10.17645/si.v6i3.1525

Calandri, E., Mastrokoukou, S., Marchisio, C., Monchietto, A., & Graziano, F. (2025). Teacher emotional competence for inclusive education: A systematic review. *Behavioral Sciences, 15*(3), 359. https://doi.org/10.3390/bs15030359

Dinnen, H. L., Litvitskiy, N. S., & Flaspohler, P. D. (2024). Effective teacher professional development for school-based mental health promotion: A review of the literature. *Behavioral Sciences,14*(9), 780. https://doi.org/10.3390/bs14090780

Fernandes, P., Jardim, J., & De Sousa Lopes, M. (2021). The soft skills of special education teachers: Evidence from the literature. *Education Sciences, 11*(3), 125. https://doi.org/10.3390/educsci11030125

Hillborg, H., Lövgren, V., Bejerholm, U., & Rosenberg, D. (2021). Integrating interventions that can support a career-oriented recovery for young adults. Building on the supported education knowledge base. *Journal of Psychosocial Rehabilitation and Mental Health, 8*(1), 35–60. https://doi.org/10.1007/s40737-020-00197-2

Hoover, S., & Bostic, J. (2021). Schools as a vital component of the child and adolescent mental health system. *Psychiatric Services, 72*(1), 37–48. https://doi.org/10.1176/appi.ps.201900575

Iahn-Aun, M., Aun, M. V., & Pereira, W. O. (2025). Characteristic competencies for effective teachers in healthcare education. *Einstein (Sao Paulo, Brazil)*, *23*, eRW1360. https://doi.org/10.31744/einstein_journal/2025rw1360

Kazanowski, Z., Żyta, A., Ćwirynkało, K., & Przybyliński, S. (2025). Teaching students with disabilities: Perceived teacher competences. *Przegląd Badań Edukacyjnych (Educational Studies Review), 2*(47), 83–106. https://doi.org/10.12775/pbe.2024.017

Lawson, G. M., Orapallo, A., Ginsburg, G. S., Brewton, A., Baker, C. N., & Azad, G. (2025). Teacher-delivered mental health interventions: Promises, challenges, and recommendations for future directions. *Adm Policy Ment Health, 52*(6), 1296–1310. https://doi.org/10.1007/s10488-025-01467-6

Lövgren, V., Hillborg, H., Bejerholm, U., & Rosenberg, D. (2020). Supported education in a Swedish context. Opportunities and challenges for developing career-oriented support for young adults with mental health problems. *Scandinavian Journal of Disability Research, 22*(1), 1–11. https://sjdr.se/articles/10.16993/sjdr.648

Manjari, A., & Srivastava, A. (2020). Teachers and schools as change agents in improving mental health among adolescents. *The International Journal of Indian Psychology, 8*(1). https://doi.org/10.25215/0801.092

Metsäpelto, R.-L., Poikkeus, A.-M., Heikkilä, M., Husu, J., Laine, A., Lappalainen, K., Lähteenmäki, M., Mikkilä-Erdmann, M., Warinowski, A., Iiskala, T., Hangelin, S., Harmoinen, S., Holmström, A., Kyrö-Ämmälä, O., Lehesvuori, S., Mankki, V., & Suvilehto, P. (2022). A multidimensional adapted process model of teaching. *Educational Assessment, Evaluation and Accountability, 34*(2), 143–172. https://doi.org/10.1007/s11092-021-09373-9

Paakkari, O., Kulmala, M., Lyyra, N., Saaranen, T., Lindfors, P., & Tyrväinen, H. (2024). The core competencies of a health education teacher. *Health Promotion International, 39*. https://doi.org/10.1093/heapro/daae078

Prabhu, S. G., Mallikarjun, P. K., Palmer, A., Nag, R., Khadeparkar, P., Arelingaiah, M., Lakshman, K. A., Podiya, J. K., Navaneetham, J., & Hugh-Jones, S. (2025). Mental health literacy in secondary school teachers and interventions to improve it – A systematic review and narrative synthesis. *J Ment Health, 34*(4), 476–495. https://doi.org/10.1080/09638237.2024.2426994

Richter, A., Sjunnestrand, M., Romare Strandh, M., & Hasson, H. (2022). Implementing school-based mental health services: A scoping review of the literature summarizing the factors that affect implementation. *International Journal of Environmental Research and Public Health, 19*(6), 3489. https://doi.org/10.3390/ijerph19063489

Suwanwong, C., Jansem, A., Intarakamhang, U., Prasittichok, P., Tuntivivat, S., Chuenphittayavut, K., Le, K., & Lien, L. (2024). Modifiable predictors of mental health literacy in the educational context: A systematic review and meta-analysis. *BMC Psychology, 12*, 378. https://doi.org/10.1186/s40359-024-01878-4

Woolf, S. (2018). Critical skills for special educator effectiveness: Which ones matter most, and to whom? *Teacher Education and Special Education, 42*, 132–146. https://doi.org/10.1177/0888406418776714

Zakaria, M., Huey, C., Suhairom, N., Saud, M., & Salleh, S. (2024). Competencies elements for vocational teachers of learners with special educational needs: A systematic literature review analysis. *Journal of ICSAR*, *8*(2). https://doi.org/10.17977/um005v8i2p327

Äärelä, T., Määttä, I., & Uusiautti, S. (2016). Ten encounters between students and a special education teacher at a Finnish hospital school – Outlining hospital school pedagogy. *Global Journal of Human-Social Science Research, 16*, 9–20. http://socialscienceresearch.org/index.php/GJHSS/article/download/1731/1672
